# Enhancement mechanisms of short-time aerobic digestion for waste activated sludge in the presence of cocoamidopropyl betaine

**DOI:** 10.1038/s41598-017-13223-4

**Published:** 2017-10-18

**Authors:** Siqing Xia, Yun Zhou, Everett Eustance, Zhiqiang Zhang

**Affiliations:** 1 State Key Laboratory of Pollution Control and Resource Reuse, College of Environmental Science and Engineering, Tongji University, Shanghai, 200092 China; 2 Biodesign Swette Center for Environmental Biotechnology, Arizona State University, Tempe, AZ 85287-5701 USA

## Abstract

Cocoamidopropyl betaine (CAPB), which is a biodegradable ampholytic surfactant, has recently been found to dramatically enhance the aerobic digestion of waste activated sludge (WAS) in short-time aerobic digestion (STAD) systems. Therefore, it is important to understand the mechanisms in which CAPB enhances WAS aerobic digestion performance. Results showed that CAPB could dramatically enhance the solubilization of soluble proteins (PN), polysaccharides (PS), nucleic acids (NA) and humic-like substances (HS) in the STAD system within the initial 2 h. Then PN, PS and NA gradually decreased, while HS showed only minor decease. In addition, CAPB increased the proportion of low MW fractions (<20 kDa) from 4.22% to 39.4%, which are more biodegradable. Specific oxygen uptake rates and dehydrogenase enzyme activity results indicated that CAPB markedly improved the aerobic microorganism activities. Microbial community analyses and principle coordinate analyses (PCoA) revealed that CAPB increased the proportion of some functional microorganisms, including *Proteobacteria, Planctomycetales*, *Acinetobacter*, *Pseudomonas* and *Aeromonas*. The changes driven by CAPB could explain the enhanced performance of the STAD system for WAS aerobic treatment.

## Introduction

As an important treatment method of waste activated sludge (WAS), aerobic digestion has been widely applied in middle and small wastewater treatment plants (WWTPs)^1–3^. In the digestion process, endogenous respiration prevails over bacterial growth due to the shortage of carbon supply. Additionally, some organisms serve as a carbon source through cellular lysis^4^. Accordingly, the degradable fraction of WAS continuously decreases and the residual fraction, which is low in energy content, is considered biologically stable^5,6^. In addition, aerobic digestion could also significantly affect WAS properties and features, including floc size, settleability, flocculability, dewaterability, aerobic microorganism activity and community speciation^2,3^. Compared with the traditional long-time aerobic digestion system, the short-time aerobic digestion (STAD) system can quickly stabilize WAS and markedly decrease the demand for oxygen^3,7^.

Surfactants are commonly used in household detergents, industrial and institutional cleaners, personal care products, floatation and petroleum production, and cosmetics all over the world due to their excellent dispersive properties and relatively low cost^8^. Most of the surfactants are discarded into the municipal sewer systems ending up at WWTPs. The biodegradable surfactants can be removed by a combination of adsorption and biodegradation by activated sludge during the wastewater treatment process^9^.

The surfactants adsorbed in WAS, however, can influence the sludge properties, which effects the aerobic digestion of WAS. Firstly, surfactants can change the dewaterability and settleability of WAS. Chen *et al*.^10^ reported that the water content decreased by about 2% as a 0.1 g cocoamidopropyl betaine (CAPB) was employed, but does not decrease further with increasing surfactant dosage. After settling for 30 min, the sludge volume was 96, 85 and 80.1 ml for the blank test, pH 2.5 and pH 2.5 plus surfactant, respectively. The explanation for surfactant-enhancing sludge dewatering and settling was the solubilization. The non-polar alkyl chains in CAPB formed micelles. which increased the aqueous solubility and accelerated the solubilization of WAS^11^. The reduction of biopolymers resulted in a more compact activated sludge under the same mechanical force. Secondly, surfactant can change the microbial community in WAS. Lozada *et al*.^12^ studied the effects of nonylphenol ethoxylate (NPEO) surfactants on the bacterial diversity in lab-scale activated sludge reactors. *Betaproteobacteria*, accounted for up to one-third of 4′,6-diamidino-2-phenylindol- dihydrochloride (DAPI)-stained cells in NPEO amended reactors, while only 5% of DAPI-stained cells in the control. The parallel abundance of unique phylotypes in reactors implied a distinctive selection of dominant organisms, which are better adapted to specialized niches in the highly selective environment.

CAPB, as a nontoxic and biodegradable ampholytic surfactant, is widely used in personal care products and surface cleaners^13^, which can be directly discharged to municipal WWTP. WAS produced from municipal WWTPs contain CAPB that may influence the aerobic treatment of waste activated sludge. According to our new findings^7^, in the presence of CAPB, the removal efficiency of volatile suspended solids (VSS) by the STAD system was up to 28.3% within 24 h, and the biodegradation rate constants of both VSS and TCOD increased by over 65% (Figure S1). CAPB can be biodegraded by the system, and the removal efficiency at 24 h was 91.2% (Figure S2). Thus, it is of big significance for WAS aerobic treatment to further disclose the enhancement mechanisms.

The purpose of this study was to investigate the effects and mechanism of CAPB in the STAD process for WAS from various perspectives, including the analysis of soluble biopolymers (concentrations, excitation-emission matrix (EEM) spectra and molecular weight (MW) distribution), the aerobic microorganism activities (specific oxygen uptake rate (SOUR) and dehydrogenase enzyme activity (DHA)), and the microbial community.

## Results

### Effects of CAPB on biopolymer release of WAS in the STAD system

**Figure 1:**
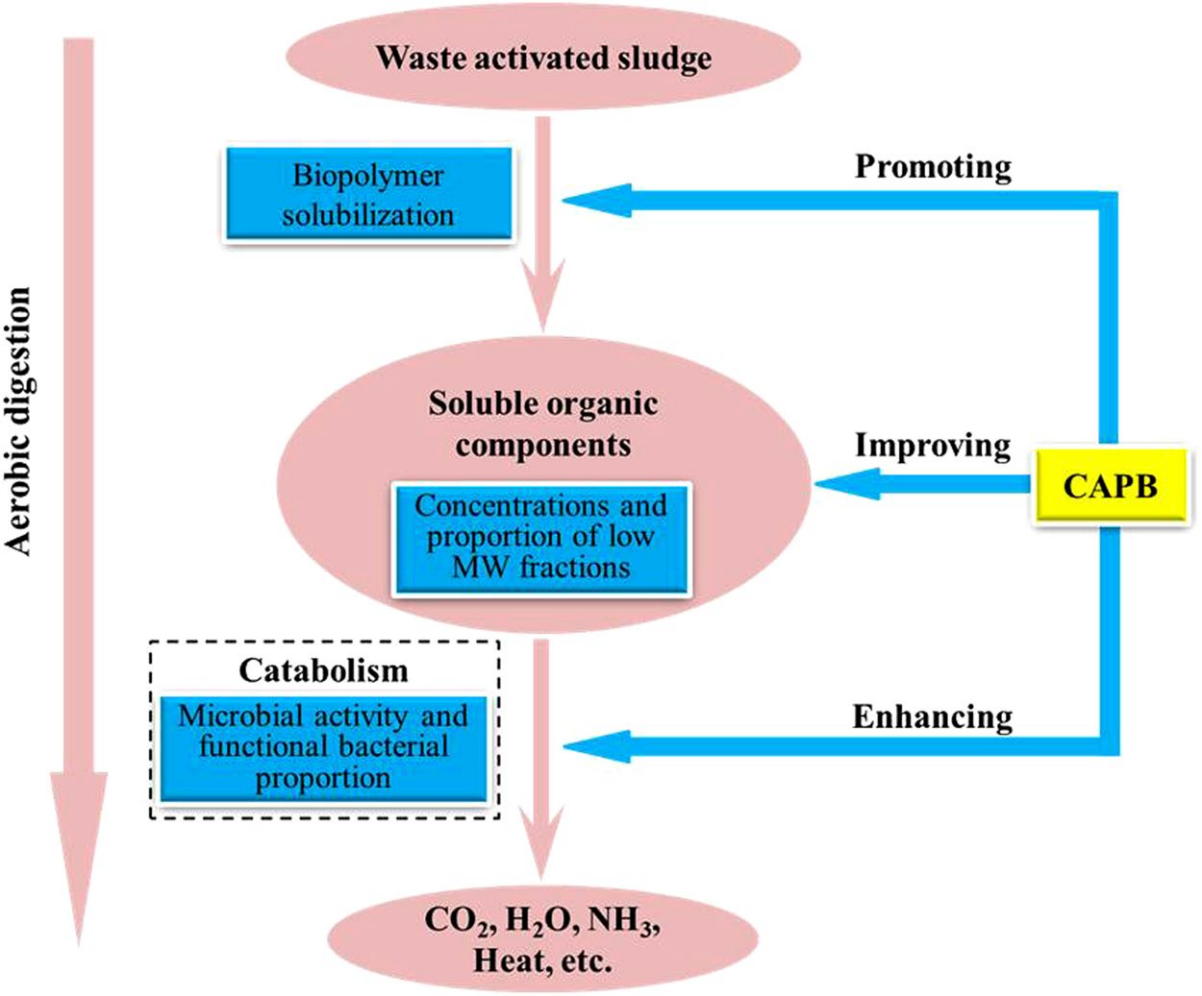
Variations of soluble proteins, polysaccharides and nucleic acids in the supernatant with and without adding CAPB during the STAD process.

Figure 1 shows the variation of soluble proteins (PN), polysaccharides (PS), and nucleic acids (NA) in the supernatant at the noted STAD times. Without CAPB, the concentrations of soluble PN, PS and NA gradually decreased in the first 8 h, as they are used as carbon and electron-donor substrates for heterotrophic bacteria^14^. The shortage of biodegradable carbon sources during longer digestion times, however, caused the hydrolysis of EPS and possibly cell lysis^2,15^, which resulted in the increase of PN, PS and NA concentrations in the longer STAD process times. By adding CAPB, the soluble PN, PS and NA concentrations drastically increased by roughly 15.1, 4.76 and 14.9 times, respectively, compared with the control experiment of WAS digested for 2 h. CAPB has a non-polar alkyl chain, which can form micelles and increase the solubility of biopolymers in aqueous solution^11^.

Therefore, CAPB can enhance the biopolymer solubilization, which results in the increase of soluble PN, PS and NA concentrations in the initial stage^10^. The concentration of soluble PN in the supernatant was higher than that of PS and NA, denoting that PN was the predominant component in the biopolymers^3^. The concentrations of PN, PS and NA decreased as the WAS digestion time increased from 2 to 24 h, which indicates the degradation of biopolymers by hydrolytic enzymes^16^.

### Effects of CAPB on 3D-EEM spectra of the supernatant in the STAD system

**Figure 2:**
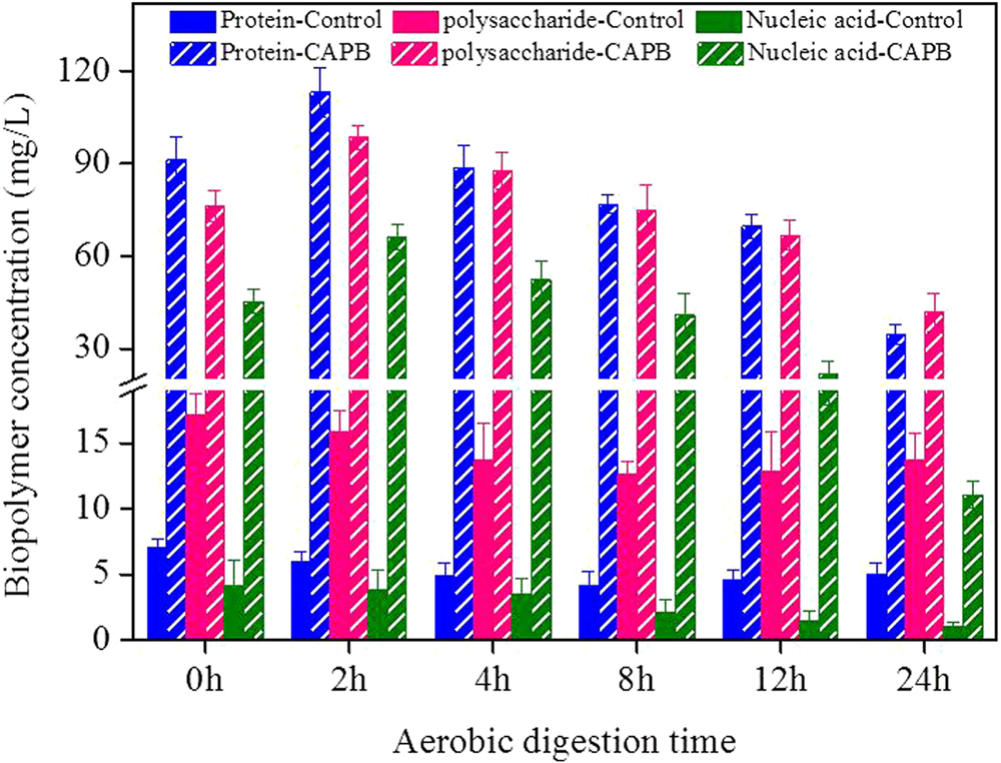
Variations of fluorescence EEM spectra and the fluorescence intensities of peak (**a**–**c**) of the biopolymers in the supernatant without and with adding CAPB at the noted STAD times. The left two figures (**a** and **c**) represent the control experiment and the right two figures (**b** and **d**) represent the experiment results after adding CAPB).

Figure 2 (a and b) show the 3D-EEM fluorescence spectra of the biopolymers in the supernatant with and without CAPB at the noted STAD times. Three main peaks were identified from the fluorescence spectra. Peak A, with the Excitation (Ex)/Emission (Em) of 310–360/410–450 nm, was associated with humic-like substances (HS). Peaks B and C were associated with the aromatic and tryptophan protein-like substances with the Ex/Em of 220–230/320–340 nm and 270–290/320–340 nm, respectively^17,18^.

Figure 2 (c and d) shows the fluorescence intensities of peaks A-C with and without CAPB at the noted STAD times. Without adding CAPB, the fluorescence intensities of peaks B decreased about 61.1%, while peaks A and C showed a minimal decrease until 12 h of digestion, but from 12–24 h all three peaks showed a slight increase. Thus, PN are more readily biodegraded compared with HS in aerobic digestion processes^19^. However, when adding CAPB, the fluorescence intensities of peaks A, B and C rapidly increased in the initial 2 h of the digestion, followed by a steady decrease throughout the remain digestion time. Tryptophan protein-like substances (peak C) showed the largest change from the control group, by having a significantly higher concentration, while HS (peak A) and aromatic protein-like substances (peak B) showed smaller changes but similar trends to peak C. This suggests that CAPB could enhance the release of PN and HS in the initial stage of the STAD process allowing proteins to be gradually biodegraded, while HS showed minimal degradation using the STAD process. CAPB improved the solubilization of the biopolymer and even caused cell lysis, which might lead to a decrease in the functional bacteria for the biodegradation of aromatic substances accounting for a decreased removal efficiency of aromatic substances^11^.

### Effects of CAPB on MW distribution of the supernatant in the STAD system

**Figure 3:**
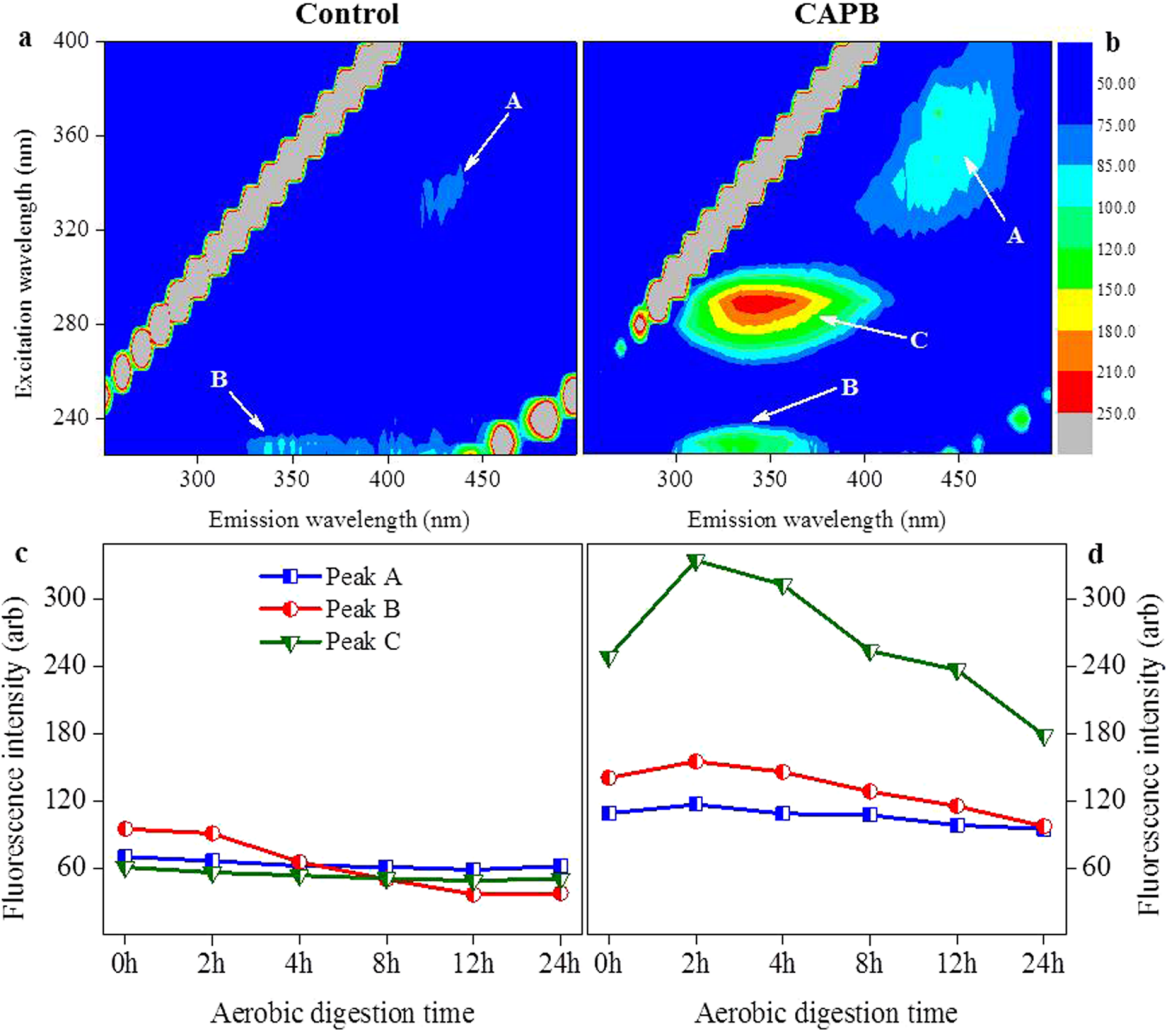
MW distributions (**a** and **b**) and the percentages of MW distributions (**c** and **d**) of the biopolymers in the supernatant without (left) and with (right) adding CAPB during the STAD process.

The biopolymer degradation by heterotrophic bacteria is influenced by its molecular weight distribution^20^. Figure 3 shows the MW distribution in the supernatant and the control showed several peaks of MW distribution. The fraction of MW > 20 kDa was dominant in the supernatant, ranging from 87.4% to 98.4% throughout the STAD process. In addition, the specific intensity of the fractions with the MW from 20 to 30 kDa, and 100 to 1000 Da decreased in the initial 8 h, which also confirmed the biodegradation of these fractions in the STAD system. However, both peaks shifted right and the specific intensities increased, which means that the molecules were degrading during the STAD process. When adding CAPB, the fraction of MW < 20 kDa dramatically increased, from 16.8% to 39.4%, and the specific intensity of the fractions with the MW from 10 to 20 kDa and 10 to 1000 Da dramatically increased compared with those without CAPB. This also suggests that CAPB could significantly improve the release of biopolymers, especially extracellular polymeric substances (EPS) from the WAS^10,21^. Moreover, the specific intensity of the above fractions decreased systematically with further digestion of WAS, which also proves the biodegradation of biopolymers in the STAD process^16^.

### Effects of CAPB on aerobic microbial activities in the STAD system

**Figure 4:**
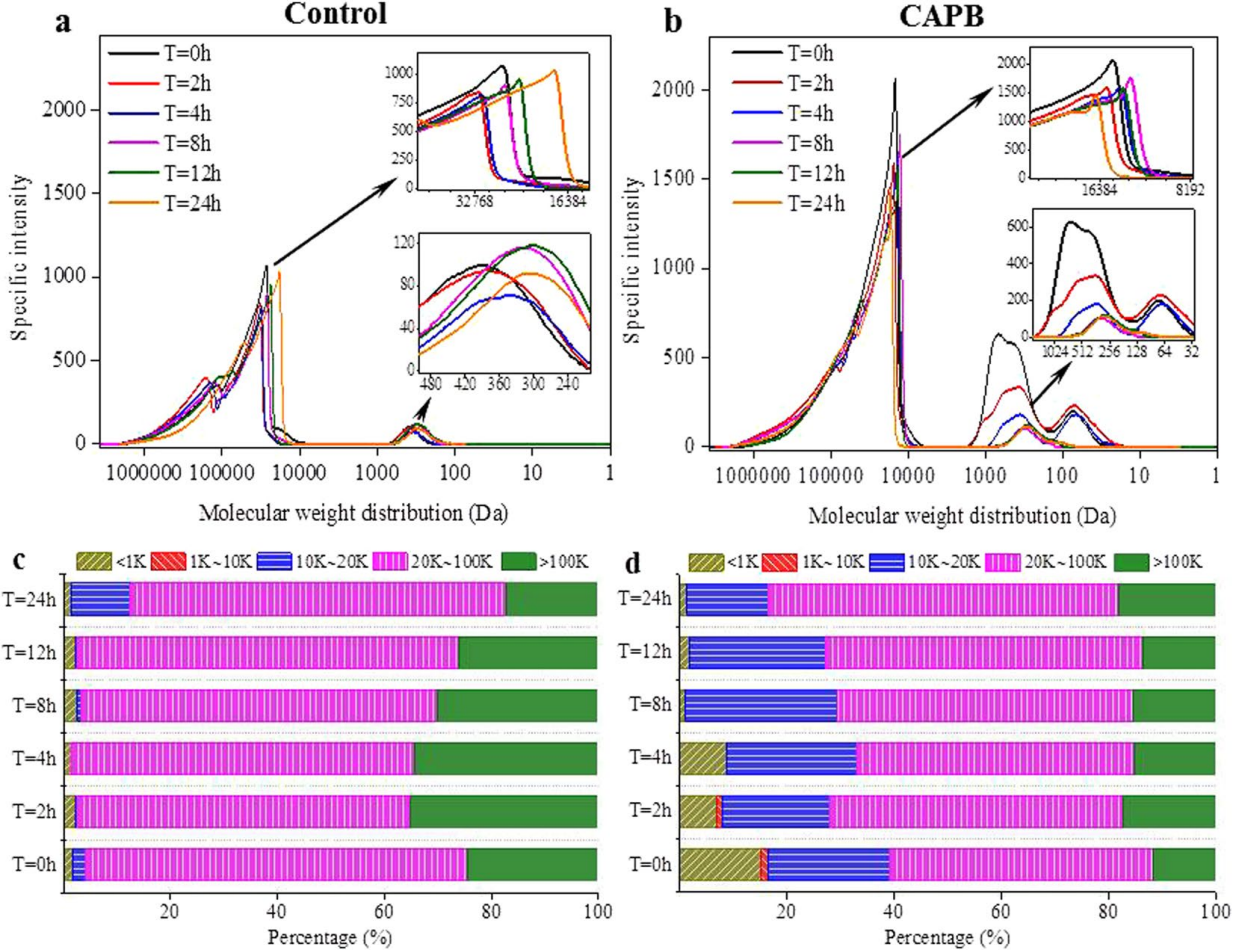
Variations of (**a**) SOUR and (**b**) TCC-DHA with and without adding CABP during the STAD process.

Biodegradation of biopolymers in WAS system is a dehydrogenation process mediated by many different intracellular and specific dehydrogenase enzymes^22^. As fundamental physiological indexes of microbial growth, both (SOUR and DHA are commonly used for evaluating the activities of aerobic microorganisms in activated sludge^23,24^. Figure 4 shows the variations of sludge SOUR and DHA with and without CAPB in the STAD system. Without CAPB, both SOUR and DHA concentrations in aerobic digested sludge rapidly decreased from 21.9 to 6.55 mg O_2_/g VSS · h and 308 to 93.2 mg TF/g VSS · h, respectively, in the initial 2 h. The significant decrease of the aerobic microorganism activities in the initial stage could be resulted from the shortage of carbon sources for microbial growth^2,15^. However, they showed a slight increase from 12 to 24 h, which could result from the supplement of carbon sources due to the releasing of soluble organic matter in the latter stage^10^. When adding CAPB, both SOUR and DHA increased immediately, and then gradually decreased throughout the STAD process. However, they were still higher than those of the control. Accordingly, CAPB could effectively improve the aerobic microorganism activities in the STAD process.

### Effects of CAPB on microbial community in the STAD system

#### Microbial community structure according to dominant classes and genera

**Figure 5:**
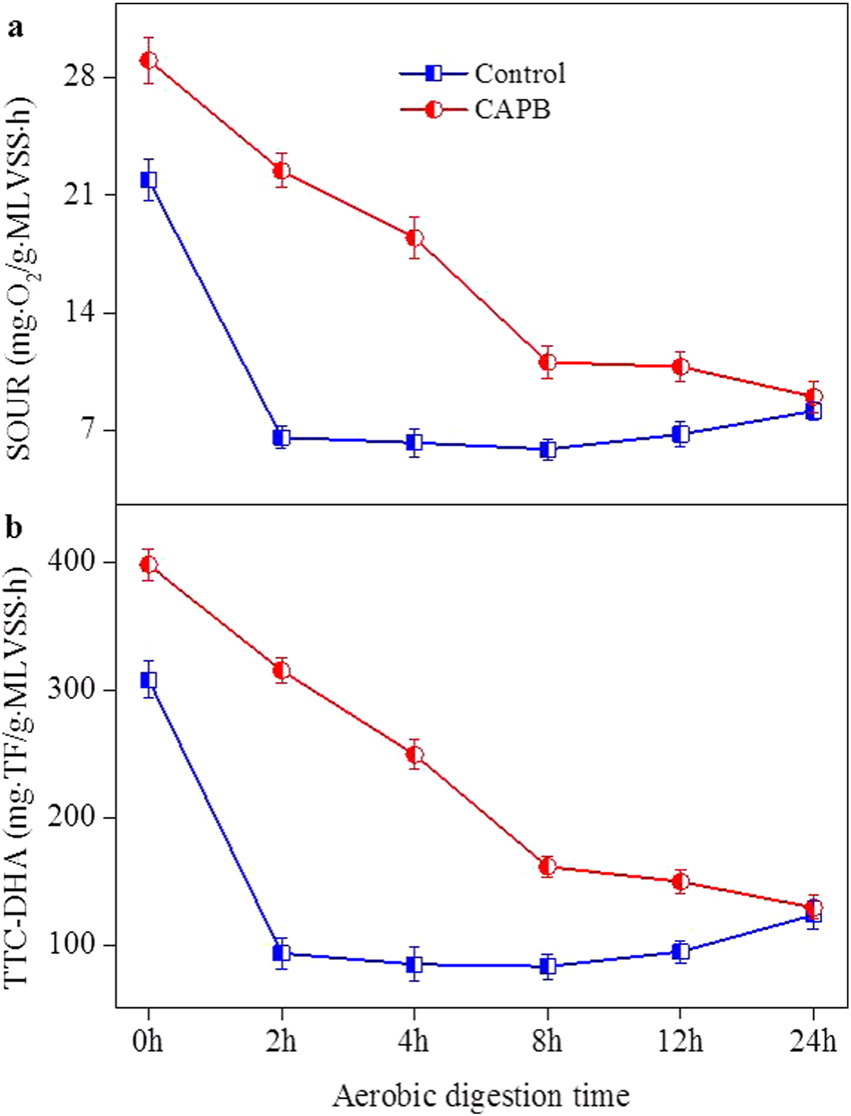
(**a**) Clustering based on the unweighted UniFrac analyses of the microbial community structure at the class level (relative abundances of dominant microbial phylotypes) and (**b**) the predominant bacterial genera in digested sludge. S1-S3 are the digested sludge in the control at 0 h, 8 h, and 12 h, respectively; S4-S5 are the digested sludge with CAPB 8 h and 12 h, respectively.

Pyrosequencing targeting the V3-V5 regions of the 16 S rRNA gene were used to analyze the diversity and structure of the WAS microbial community throughout the STAD process. Figure 5a shows the relative abundances of the most abundant phylotypes at the class level throughout the experiment. Without adding CAPB (S1~S3), the phylotypes in bacteria-rich WAS remained relatively stable in the initial 8 h, and the main bacteria were *γ-Proteobacteria* (~12%), *β-Proteobacteria* (~9.3%), *Sphingobacteria* (~22%) and *Clostridia* (~9.7%). However, phylotypes closely related to *γ-Proteobacteria* rose to 17% and *planctomycetacia* increased from 1.64% to 3.37% after 12 h. When adding CAPB (0.08 g/g dry sludge), the microbial community of WAS significantly changed in the initial 8 h (S4). *γ-Proteobacteria* became the dominant phylotypes (~65%), the abundance of *α-Proteobacteria* and *planctomycetacia* also rose to 6.2% and 4.1%, respectively. However, the abundances of other phylotypes reduced or disappeared. After aerobic digested for 12 h (S5), the abundances of *β-Proteobacteria*, *Chlorobia* and *Flavobacteria* gradually increased and reached to 9.5%, 4.7% and 5.4%, respectively. Therefore, the addition of CAPB siginificantly altered the community structure of WAS during STAD.

Figure 5b shows the relative proportions of the most abundant bacterial genera in WAS throughout the STAD process. Without CAPB, *Pseudomonas* and *Clostridium* were the dominant genera with the proportions of 5.0% and 7.5%, respectively. Throughout the STAD process (S1-S3), *Aeromonas*, *Kluyvera* and *Cloacibacterium* were barely detectable, the proportions of *Pseudomonas* remained stable, *Acinetobacter* showed a slight increase, while *Clostridium* decreased to 3.8% after digestion for 12 h (S3). When adding CAPB, the genus *Clostridium* nearly disappeared, while the proportion of *Pseudomonas* dramatically increased to 36% and became the dominant genus after 8 h (S4), but decreased in the latter stage (S5). The proportions of *Aeromonas* and *Cloacibacterium* kept increasing and reached 9.3% and 4.5% after 12 h, respectively. The proportions of *Caldilinea* and *Acinetobacter* also increased in the first 8 h, but reduced in the later stage of aerobic digestion.

*Proteobacteria* was identified as one of the bacteria responsible for the biodegradation of organic components in WAS^25^. *Planctomycetales* was identified as the bacterium responsible for the biological nitrogen removal^26^. *Acinetobacter* was identified as a predominant group of bacteria responsible for enhancing biological phosphate removal by many researchers^27^. Aerobic biodegradation of quaternary ammonium compounds (QACs) has been attributed mainly to bacterial species in the genera of *Pseudomonas* and *Aeromonas* from *γ-Proteobacteria* ^28^. Results showed that in the STAD process, CAPB could significantly improve the proportions of some functional microorganisms, including *Proteobacteria, Planctomycetales*, *Acinetobacter*, *Pseudomonas* and *Aeromonas* responsible for biological removal of biopolymers, the uptake of nitrogen and phosphorus, and the biodegradation of CAPB, respectively. Thus, the WAS stabilization efficiency was improved.

#### Forces driving the microbial community structure elucidated by PCoA

**Figure 6:**
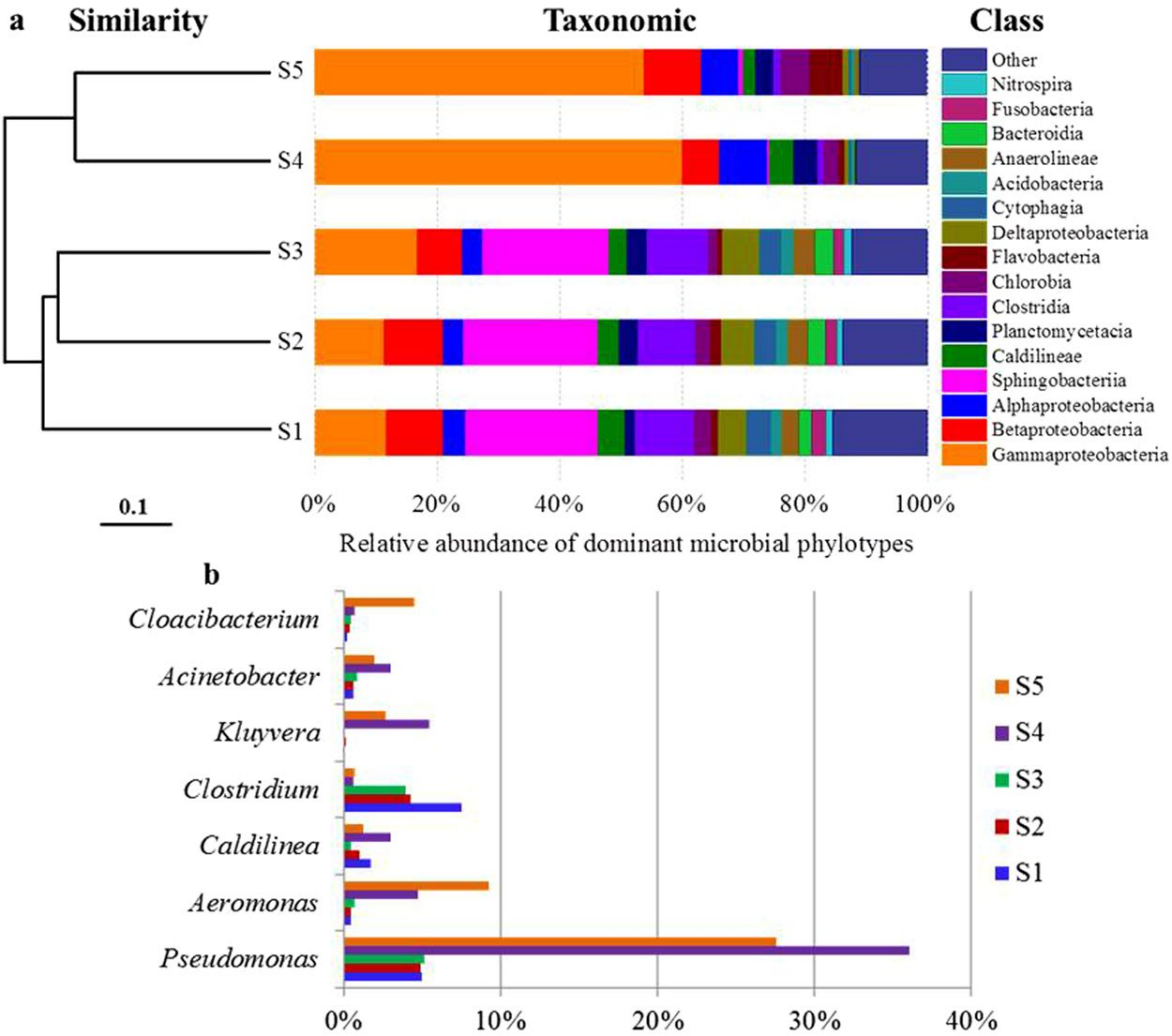
PCoA results based on the unweighted UniFrac analyses showing the microbial community grouping in activated sludge with and without adding CAPB during the STAD process.

PCoA is a method to explore and to visualize similarities and differences in the microbial communities^29^. Figure 6 shows the unweighted PCoA results based on the absence or presence of phylotypes. The most important trend was that digested sludge samples for S1-S3 (without CAPB) together have much higher PC1 values compared to those for S4-S5 (with CAPB). This further confirmed that the addition of CAPB could significantly influence the microbial community structure. In addition, the sludge samples for S4-S5 were different from each other. Therefore, CAPB could significantly alter the microbial community structure during STAD.

## Discussion

As a widely used surfactant, CAPB was found to promote the fast removal of organics in WAS and the removal efficiency of VSS increased to 28.3% within 1 day, while previous studies reported have not reported removal efficiency higher than 20%. In addition, CAPB was also biodegraded by the system with removal efficiency of 91.2% after aerobic digestion for 1 day. Soluble biopolymers and their 3D-EEM spectra analyses showed that in the presence of CAPB, the concentrations of soluble PN, PS, NA and HS in the STAD system rapidly increased within the initial 2 h. Then PN, PS and NA gradually decreased, while HS showed a minimal decrease. From the results of molecular weight (MW) distributions analyses, CAPB increased the fraction (from 4.22% to 39.4%) of low MW fractions (<20 kDa), which were more readily biodegraded. The analyses of both SOUR and DHA indicated that the aerobic microorganism activities in the STAD system were markedly improved in the presence of CAPB. According to the microbial community analyses and PCoA, the presence of CAPB caused in increase in certain functional microorganisms, including *Proteobacteria, Planctomycetales*, *Acinetobacter*, *Pseudomonas* and *Aeromonas*. The changes driven by CAPB could explain the enhanced performance of the STAD system for WAS aerobic treatment.

**Figure 7:**
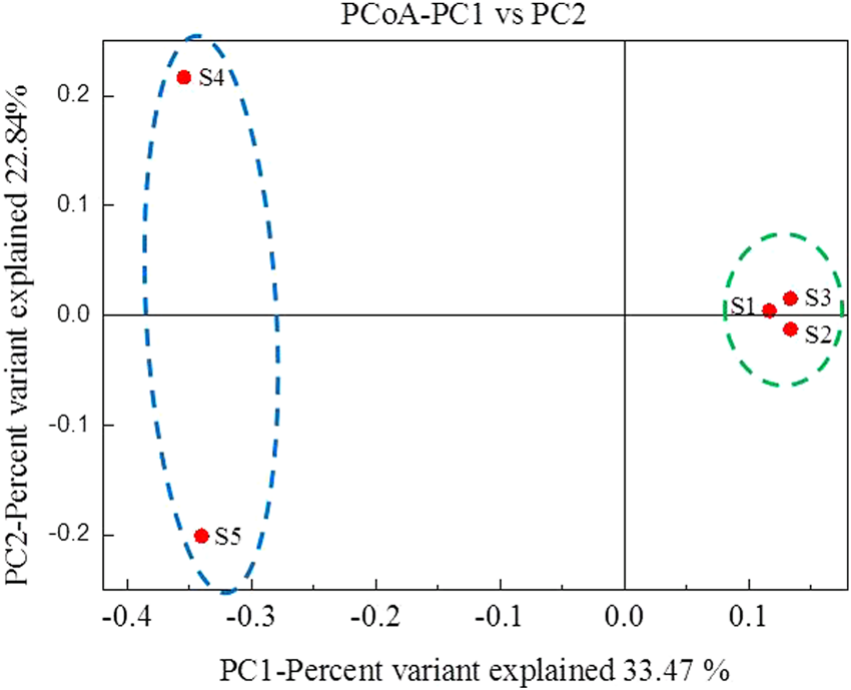
Enhancement mechanisms of short-time aerobic digestion for waste activated sludge in the presence of CAPB.

In conclusion, as a widely used ampholytic surfactant, CAPB could dramatically enhance the aerobic digestion of waste activated sludge (WAS) in the STAD system. The proposed mechanisms of CAPB include the following three aspects (Fig. 7): promoting the solubilization of biopolymers; improving the concentrations and fraction of low MW products, which are readily biodegraded; enhancing the microbial activity and the proportion of functional bacteria. Thus, the combination of short-time aerobic digestion and CAPB could be a promising method for the WAS reduction treatment and the biodegradable CAPB could lead to a promising performance of the STAD process for WAS and resulted in zero waste discharge from the treatment system.

## Materials and Methods

### Chemicals and sludge samples

CAPB surfactant, with a molecular weight of 342.52 g/mol, was obtained from Shanghai Chem. Co. Ltd., China. The WAS used in this study was obtained from the secondary settling tank of a full-scale municipal WWTP in Shanghai, China. The collected sludge samples were transported to our laboratory within 30 min. Sludge samples were subsequently screened through a 1.2 mm sieve to remove grit and then concentrated by setting at 4 °C for 2 h. The main parameters of concentrated sludge were as following: pH 6.8 ± 0.3, total suspended solids (TSS) 8.4 ± 0.4 g/L, and VSS 5.8 ± 0.6 g/L. Concentrated sludge was stored at 4 °C and used within 2 days.

### Aerobic digestion of WAS and sample collection

Experiments of CAPB influence on the STAD of WAS were carried out in two identical reactors, which were made of plastic cylinders with a volume of 6.0 L (Φ12 cm × 53 cm) with a working volume of 5.0 L and placed on an accurate strengthen electronic stirrer (JJ-1, Changzhou, China) with blades for mixing the samples at 350 rpm (Figure S3). The experiments were maintained at room temperature (25 ± 1 °C) using a KFRd-120L/BAC13 air conditioner, and the dissolved oxygen (DO) was maintained at 2–3 mg/L using a microporous aeration disk at the bottom of the reactor. CAPB was added to one reactor with the dosage of 0.08 g/g dry sludge according to prior optimization results^7^. The other reactor without adding CAPB was the control. Sludge samples collected from both reactors throughout the experiment were immediately characterized. Before collecting samples each time, some deionized water was added to supply the evaporation losses of the culture. Part of the sludge sample from each reactor was centrifuged at 4000 × *g* for 20 min at 4 °C using a high-speed freezing centrifuge (Heraeus Multifuge × 1 R, Thermo Scientific, Germany). The supernatant was collected and stored at 4 °C. Prior to the analyses of liquid phase, the supernatant was centrifuged at 12000 × *g* for 10 min to further remove particles. Part of the sludge sample was directly used for the analyses of SOUR and DHA of the digested sludge. Part of the sludge sample stored at −20 °C prior to the microbial community analyses.

### Chemical analyses

The PN in the supernatant was determined by the Bradford method using bovine serum albumin as the standard^30^. The PS in the supernatant was measured by the phenol-sulfuric acid method using glucose as the standard^30^. The NA in the supernatant was determined by the diphenylamine colorimetric method using calf thymus deoxyribonucleic acid as the standard^30^. The fluorescence spectra of the supernatant was determined by a 3D-EEM fluorescence spectrophotometer (Varian Cary Eclipse, Agilent, Australia) equipped with a Xenon lamp as the excitation source. The MW distribution of the biopolymer in supernatant was analyzed using a high-performance size exclusion chromatography (LC-10AD, Shimadzu, Japan). For the determination of the oxygen uptake rate (OUR) of WAS, aerators and probe of dissolved oxygen meter (HQ40d, HACH, USA) were placed in the sealed glass bottle with the volume of 100 ml. Sludge samples in the bottle were aerated for 5 min to saturate with oxygen saturated and then stopped. The DO concentration was measured by the DO meter and continuously monitored by a computer. The OUR of sludge sample was calculated by a linear regression analysis and then quantified to SOUR based on VSS^31^. The DHA of digested sludge was measured by the TTC-reduction method using triphenyltetrazolium chloride (TTC) as the indicator^32^.

### Microbial community analyses

For the DNA extractions, sludge samples in both STAD reactors were collected with the aerobic digestion time of 0, 8, and 12 h. 10 mL of sludge sample was centrifuged at 12000 × *g* for 20 min at 4 °C. The supernatant were removed and the pellets were resuspended using PBS buffer (molar ratio of Na_3_PO_4_, Na_2_HPO_4_, NaCl, and KCl is 2:4:9:1)^33^. The total genomic DNA of each sample was extracted using Fast DNA^®^ Spin Kit for Soil (MP Biomedicals, USA) following the manufacturer’s instructions. DNA samples were sent to Beijing Genewiz biological technology (Beijing, China) to perform sequencing library preparations and standard Illumina MiSeq sequencing protocols. The forward primers containing the sequence “CCTACGGRRB GCASCAGKVRVGAAT” and reverse primers containing the sequence “GGACTAC NVGGGTWTCTAATCC” were used to amplify the V3 and V4 regions, while the forward primers containing the sequence “GTGYCAGCMGCCGCGGTAA” and reverse primers containing the sequence “CTTGTGCGGKCCCCCGYCAATTC” were used to amplify the V4 and V5 regions of bacterial 16 S rRNA genes. DNA libraries were validated using an Agilent 2100 Bioanalyzer (Agilent Technologies, Palo Alto, CA, USA), and quantified by Qubit and real time PCR (Applied Biosystems, Carlsbad, CA, USA). DNA libraries were multiplexed and loaded on an Illumina MiSeq instrument (Illumina, San Diego, CA, USA) according to manufacturer’s instructions. Sequencing was performed using a 2 × 250 paired-end (PE) configuration; Image analysis and base calling were conducted by the MiSeq Control Software (MCS) on the MiSeq instrument. We evaluated the overall community using the unweighted UniFrac distance matrix^34^ and the relationships among samples with Cytoscape and principle coordinate analysis (PCoA)^35^. All the 16 S rRNA gene-sequencing data were analyzed using the QIIME 1.7 software as described by Lai *et al*.^36^.

## Electronic supplementary material

Supplementary Information
