# Supplementary material for: Enhancement mechanisms of short-time aerobic digestion for waste activated sludge in the presence of cocoamidopropyl betaine
Source: Sci Rep. 2017 Oct 18;7:13491. doi: 10.1038/s41598-017-13223-4 (PMC5647449; doi:10.1038/s41598-017-13223-4)
Supplement: Supplementary file 1 — Supplementary Information [file 41598_2017_13223_MOESM1_ESM.pdf]

Supplementary Information

Enhancement mechanisms of short-time aerobic digestion for waste activated sludge in the presence of cocoamidopropyl betaine

Siqing Xia [1], Yun Zhou [1, 2], Everett Eustance [2], Zhiqiang Zhang [1, *]

[1] State Key Laboratory of Pollution Control and Resource Reuse, College of Environmental Science and Engineering, Tongji University, Shanghai 200092, China

[2] Biodesign Swette Center for Environmental Biotechnology, Arizona State University, Tempe, AZ 85287-5701, USA

* Corresponding author. E-mail address: zhiqiang@tongji.edu.cn (Z.Q. Zhang)

Determination of CAPB

The determination of CAPB in aqueous phase was based on the acid orange II method described by Wu et al[1]. Aqueous and sludge phases were separated by centrifugation at 4000 rpm for 20 min. 50 ml of aqueous sample was added into the separating funnel with the volume of 200 ml, and then mixed with 10 ml buffer solution (consisting of 97 ml of 0.2M HCl, 50 ml of 0.2M KCl and 53 ml deionized water) with the pH of 1.0 and 3 ml acid orange II/ deionized water (0.1:100, m/m). After further adding 20 ml of chloroform, the mixture solution was reacted with the hand shake for 3 min and then let it sit for 15 min. The chloroform at the bottom of the separating funnel was transferred into a 50 ml volumetric flask but without water. 20 ml of chloroform was added into the separating funnel and then further extracted the residual CAPB in the water. All of the chloroform extract were collected in the volumetric flask and diluted to its scale using chloroform. 50 ml of deionized water also was added into the separating funnel and then use the same extraction method as a control. Wet sludge samples were dried at 103 ^{o}C and then smashed, followed by Soxhlet extracted with 50 ml ethanol for 12 h. The ethanol extract, containing the CAPB, was evaporated to dryness and then redissolved in aqueous sample, and the extraction method of CAPB in the aqueous sample were shown above. Determination was performed by a UV-vis spectrophotometry (UV2600, Shimadzu, Japan) with the wavelength of 485 nm. A seven-point calibration curve was made from CAPB solutions at concentration levels between 0 and 12 mg/L, and the recoveries ranged from 86% to 97% for the CAPB.

Determination of VSS and TSS

TSS and VSS of WAS were analyzed following the standard methods[2]. As the residual CAPB could be part of VSS, the VSS of WAS could be calculated using the equations (S1).

$$VSS_{WAS} = VSS_{Total} - C_{CAPB\text{-}sludge} \quad (S1)$$

where VSS_{WAS} is the concentration of VSS of WAS; VSS_{Total} is the total concentration of VSS; $C_{CAPB\text{-}sludge}$ is the residual concentration of CAPB in sludge phase.

References

1. Wu, Y.; Ding, W.; Jiang, Y., Research progress of the content determination of effective substance of amphoteric surfactants. *J. Chem. Ind. Eng.* **1**, 025(2014).
2. American Public Health, A.; American Water Works, A.; Water Pollution Control, F.; Water Environment, F., *Standard methods for the examination of water and wastewater*. American Public Health Association. **2** (1915).

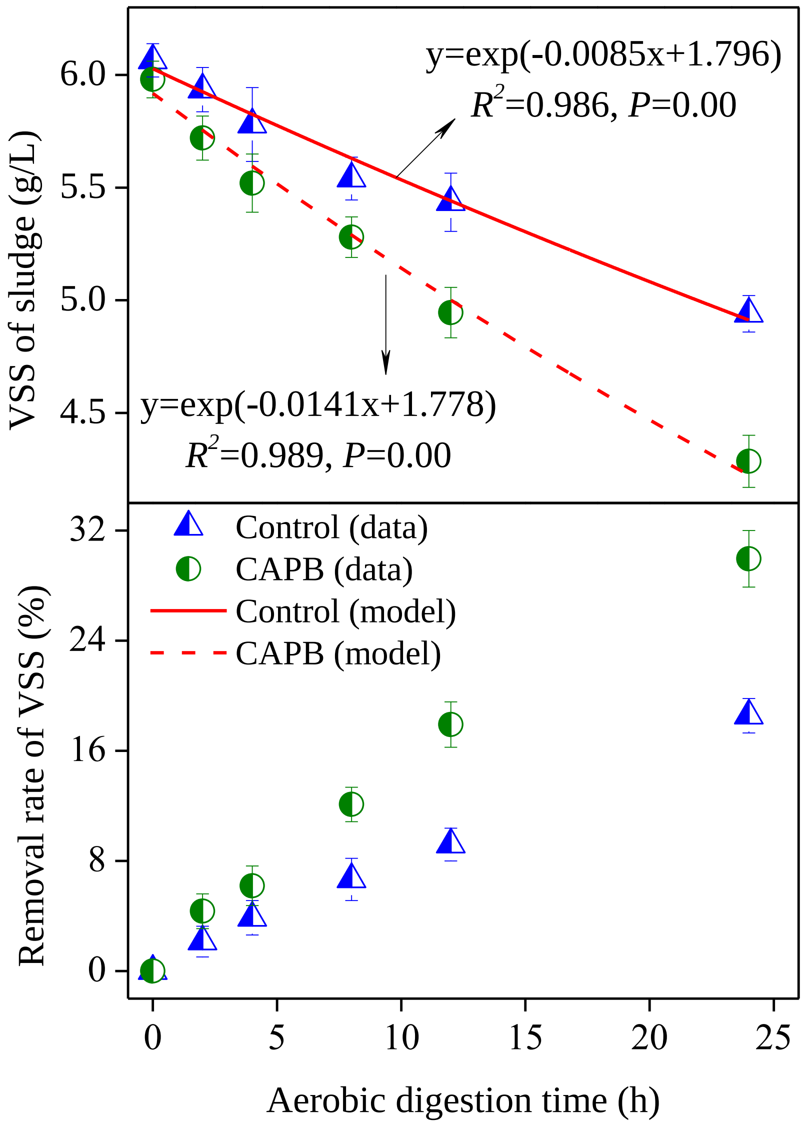

Figure S1. Concentration of VSS and its removal rate without and with adding CAPB during the STAD process (dot: measured data; line: simulated).

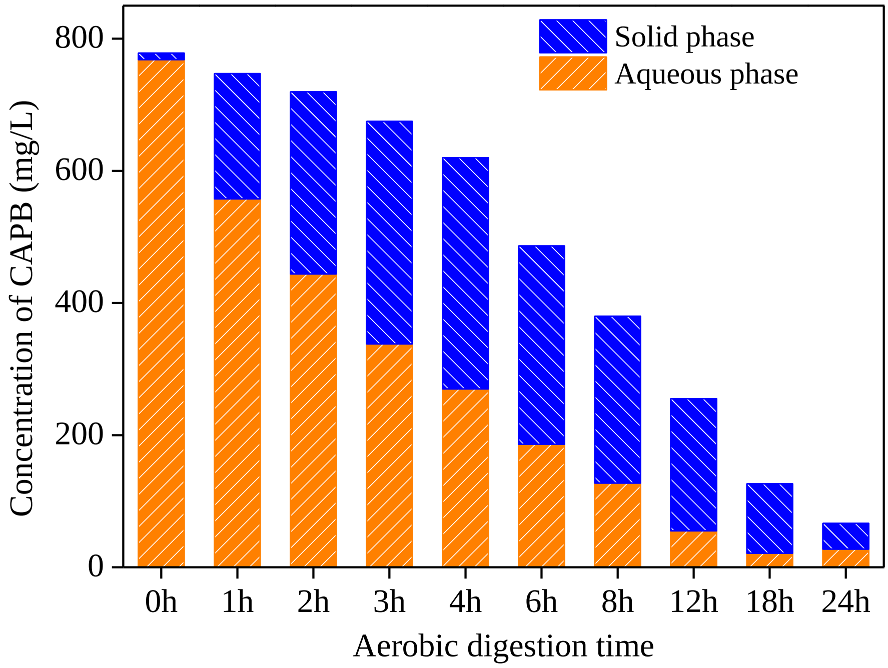

Figure S2. Variation of CAPB concentrations in aqueous and sludge phase, and its biodegradation during the STAD process.

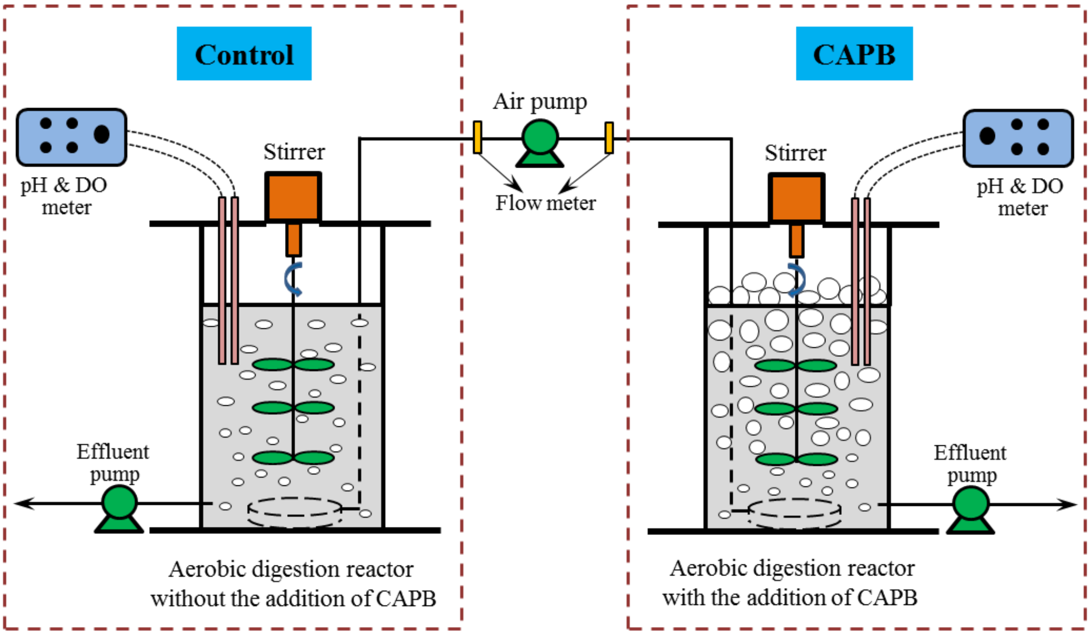

Figure S3. Schematic configuration of the lab-scale short-time aerobic digestion process without and with the adding of CAPB.
